# Prof. Dr. Liew Su-May’s Inaugural Lecture

**DOI:** 10.51866/mol.1135

**Published:** 2026-03-06

**Authors:** Jeffery Freddy Abdullah Saini

**Affiliations:** 1 KPJ KL Rehabilitation Centre, Tawakkal Health Centre, 202A, Jalan Pahang, Kuala Lumpur, Malaysia.

**Keywords:** Patient-centred care, Patient-public involvement, Family physician

Prof. Dr. Liew Su-May, former Chief Editor of the Malaysian Family Physician passed away in December 2021. She was due to present her inaugural lecture at Universiti Malaya in 2022 after having surgery for her bladder cancer. Complications from her surgery and subsequent antiphospholipid syndrome complications meant that it was not to be.

I forgot that she had sent this to my Google Drive to read through after she had written it prior to her surgery and found it by accident when I was looking for something else. It was as if she was nudging me to present it, so I presented this during the Dr Liew Su-May Research Award in December last year at her department.

The current editor thought that it would be a good idea to include this lecture in the Malaysian Family Physician as it chronicles my late wife’s struggles with her illness and how it shaped her towards making patient-centred care and patient-public involvement the goals of her research and care for patients. If this article inspires present and future family physicians towards patient-centred care and patient-public involvement, then this will have at least helped fulfil what she had set out to accomplish as a family physician.

**INAUGURAL LECTURE**

My esteemed colleagues, let me start by saying that this is not going to be the usual inaugural lecture. I will not be sharing the latest results of my most recent publication, nor sharing with you the most up to date evidence in the research field. If that was your expectation, I am truly sorry. In the past one year, I have had a lot of time to think about what to say and I have thought long and hard. This is going to be a personal account of my journey towards patient centred care. It is a very personal account, and I have debated over whether this was a suitable thing to talk about. Just recently, I was asked to introduce myself to a working research group on what inspired my work. The others in the group talked about their heroes, wonderful scientists throughout the ages, their parents, works of art. And who did I say was my inspiration? It was my 24-year-old self. It was difficult to get through that 5-minute introduction and I thought to myself, if i cannot speak to that small group for that short period of time, how could i think of talking to all of you for this length of time. Then i realised how difficult it must be for others to talk about their personal journeys, for patients to tell us their stories, for us to open ourselves to scrutiny and possible criticism, disgust and worst of all, pity. It is difficult but that is why I have chosen to go ahead. I have to counter my own feelings of insecurity and shame.

When I was 23 years old, in my final year of medical school, I was diagnosed with systemic lupus syndrome. SLE is an autoimmune disease which means that my immune system was attacking my body. It is called lupus, the latin word for wolf, because of the characteristic erosive facial rash that looked like a wolf’s bite. But i never really had this rash, the butterfly rash. SLE is systematic because it can affect any organ of the disease. For example, my mum had it mainly affecting her skin - a more benign form called dermoid lupus. In order to diagnose this disease as systematic, you needed to have involvement of 2 systems. Except if it affected your kidney. Lupus nephritis class 3 or 4 was enough for you to be diagnosed as SLE because it was the most dangerous organ to be affected. When I was first diagnosed, my body was producing antibodies that were attacking my clotting system, giving me clots in my lungs and arteries. It was also affecting my kidneys causing it to leak blood cells and protein.

I was put on maximum doses of steroids and cyclophosphamide to prevent my kidneys from failing and a blood thinner warfarin, to prevent my blood from clotting. I was supposed to be on the steroid and chemo regime for only 6 months. But at the end of that, my renal function did not get better, it got worse. so it continued for another 6 months. Then another and another, until the doctors, who were my professors and my lecturers, were not really sure of what to do. So they started me on continuous oral chemotherapy. I was on that for two years. That helped to subdue the SLE but then i developed bladder cancer. This was in 1999. By that time, it had been 5 years since I was first diagnosed. i had completed my housemanship and three years of service and i was undergoing postgraduate training in primary care medicine. When i first heard about the bladder cancer, it felt like a real blow. I took a year off, underwent surgery and further chemotherapy, recuperated and resumed training.

This must all sound like an episode of Drama Minggu Ini or an Oprah Winfrey episode. It’s not the usual way to start an inaugural lecture. But i wanted to tell you that before i became a doctor, before i became a specialist, I was a patient, A member of the public, a wife, a mother, a daughter. It was only in September 2019, slightly more than a year ago, that I became a professor. First and foremost i was a patient.

I have tried to deny this part of me. So much of my life, my training, had been affected by my illness. I took my final medical school examination two months after my diagnosis. I was still on high dose steroids and cyclophosphamide then. SLE was such a big part of my life during my undergraduate and postgraduate training that I made a decision while doing my phd in oxford. i was not going to tell anyone about my illness during my studies there. I wanted to be sure that my phd was all me. That it was not defined by my disease as so much of my life had been. But now i have come to the realisation that my disease is part of me, that it has shaped my life as well as my career. Let me tell you about this journey.

Often we as doctors think that we know what patients are going through. But do we really? I’m not sure that you would ever understand some of the things that are demanded of patients unless you have gone through the experience yourself. SLE has taught me a lot of things. I’m an expert at doing investigations. I mean- i don’t want to boast but i really am good at this. I know exactly how it feels like to have a tube shoved down my esophagus, dyes poured into my blood vessels, every imaginable type of scan carried out on every part of me. I’ve had a bypass, a cystoscopy, a caesarean section. I have first hand knowledge. I have heard doctors and nurses tell patients off for not collecting samples properly. I remembered my teachers telling me about how the collection of midstream urine has to be properly - no, perfectly done. But do you know what it is like to take a mid stream urine in the patient’s toilet? It is pretty impossible without getting urine everywhere including on yourself. Sometimes when i see trainees taking informed consent for procedures, i think to myself, they know nothing. Nothing. But then i check myself because i know that the patient does not want to hear from the doctor, oh i’ve done that, piece of cake. They want to know what is going to happen, what the risks are and also hear words of comfort. That’s what’s important. Patients do not want a confessional.

And can you imagine how difficult it was for me to take steroids? The first time i had to do so, i realised what was in store for me, the Cushingoid facies, the truncal obesity, the striae, diabetes, hypertension. I could see what was in store for me, but it was like seeing through a glass darkly. It is sometimes harder to know what’s coming than to not know. When i was put on high dose prednisolone, i watched my face expand every day in the mirror until i thought that it was going to explode. My weight ballooned, stretching my skin and i thought, oh now i understand what striae is, those stretched broken skin fibres. I learnt so much about medicine throughown experiences. Frothy urine due to proteinuria looks like the froth on beer. What your periods would be like if you are on warfarin - they are, in fact, normal. How to give yourself insulin injections. How to be compliant with taking all your medications.

I also understand why patients do the things they do. Things that doctors sometimes do not understand. I understand why they refuse medication, why they refuse tests, why they do not come back for follow-up, why they get angry and why they get sad.

Just recently, after a period of time of quiet and relative peace, my body started to act up again. The bladder tumour had recurred again after 15 years. i had to go through another battery of tests and surgery. Many people have told me i am brave. But it’s not bravery, i’m no hero or warrior. I tell myself the same things that i tell my patients, just take it one step at a time. Keep your eyes looking straight ahead, breathe and just do the next thing, take the next step. When it is really difficult, or there is actual pain, i think of my daughter. I think of the prayers that all mothers say when their child gets sick. Don’t let her be ill, don’t let her feel the pain, let me be the one to bear it. And if there is karma, and if by going through this, it means that she does not have pain, then i am content. And i can bear anything. That may sound pretty melodramatic saying it out loud here like that but that viewpoint has got me through some really hard patches.

My illness has shaped me, my life and my career. It is a major reason as to why i am now standing here in front of you at my inaugural lecture. In my last year of medical school, i was admitted to the medical ward during my clinical posting. The patient in the bed next to me said, Eh semalam you kan doktor yang datang clerk saya? Sekarang masuk wad? I had no time to find a patient, read the literature and write up the case study. So my first case report was on me. I learnt about my illness by looking up the medical literature on lupus nephritis. I read the seminal studies on pulse cyclophosphamide therapy for lupus nephritis. That was difficult to read. But it started me on my way in evidence based medicine. I learnt how to use the index medicus in the medical library. I also looked up papers in journals and started reading them. I didn’t understand a lot of it and definitely not the statistics then. But that was certainly the trigger for my learning.

I also read papers that discussed the prognosis for SLE. I remember very clearly an episode early on in my disease. My parents began acting very strangely around me. They could not look at me -my dad had become very quiet and my mother looked really sad. This had been happening for a while before i noticed. I asked them, what’s wrong? And they said, we found the paper. And i said, what paper? I had printed out a study that showed that the ten year survival for SLE was now 90% and my parents found the paper and read it. My parents thought that the results meant that 90% of SLE patients would only survive for ten years. It doesn’t. It means that in ten years from diagnosis 90% of those with SLE would still be alive. That showed me how easy it was to misunderstand numbers and risks in medicine. People do not understand risks. Patients, public and even Doctors think that everyone gets better with treatment and does worse without treatment. The truth is that not everyone gets better with treatment, and not everyone does worse without treatment. Mostly what we are doing is lowering risk. In a study, there are 2 groups of patients, one of them will be the treatment group and the other the control group (usual care). Some of those in the treatment group will get better. But some in the control group will also get better. Some of those in the treatment group will not do well. And of course, some of those in the control group will not do well. When we conduct a study, we are hoping that there are more of those who do better in the treatment group than the intervention group. So i can treat a patient today, lower his blood pressure, control his sugars and his weight etc. And tomorrow he can still have a heart attack. We are not able to say that we can cure everybody, we are only able to say that we are lowering risk. Not many patients understand this, and not many doctors understand this either. This might be our coping mechanism but it also leads to misunderstandings and takes away true informed consent. I have made it a mission of mine to improve understanding about this So that people can understand research findings in makingdecisions about their treatment. Informed decisions, rather than simply doing what they have been told to do or basing treatment upon beliefs and cultural norms. Of course, this is only if they want to do so.

Looking back upon my career, i can see just how much my illness has guided me. I have worked on evidence based medicine, prognostic research, medical communication, shared decision making, health literacy and so on. I have carried out research in these areas and have experienced the other side of things as a study participant. Now i am trying to develop patient and public involvement in research in Malaysia.

What is patient and public involvement? Like patient centred care, PPi aims to put the patient and the public at the centre of decision making whether it be on decisions related to their health care or with regards to research being carried out to improve their well being. Traditionally and currently, many decisions are still being made ‘for’ or ‘on’ them. These words should be changed so that decisions are made ‘with’ or ‘by’ patients or members of the public. We often think that we know better than them ‘the patients’. But it is not just doctors, often patients think the same too and want us to make the decisions for them. But times, they are a-changing.

Patient activation started with growing disillusionment with poor quality health care around the world. Doctors and scientists were knocked off their pedestals of nobility by the horrors inflicted by members of our profession during the Holocaust. Further poor practices in clinical practice such as the Alder Hey scandals and research such as the Tuskegee syphilis study led to a growing demand for transparency and actual autonomy in decision making. There are also increasing signs of our own members of the public, Malaysians, fighting to highlight poor responses from authorities such as occurred after the Pasir Gudang toxic chemical incident and the plastic waste pollution in Jenjarom.

Why do this? Professor Dame Sally Davies the former chief medical officer for England said ‘no matter how complicated the research, or how brilliant the researcher, patients and the public always offer unique, invaluable insights. Their advice when designing, implementing and evaluating research invariably makes studies more effective, more credible and often more cost efficient as well.’ PPI should start right at the beginning - research questions should address health problems that are important to the end users. This is not the case, public funding for research correlates poorly with disease burdens [Chalmers, Glasziou. 2009] This occurs here too. For example, asthma is the number one chronic disease in children yet respiratory health is not a research priority in our country.

We have also started involving patient and public members in our research here. This started with our RESPIRE project which aimed to identify and tackle gaps in the care of respiratory diseases in 4 countries namely Malaysia, India, Bangladesh and Pakistan. RESPIRE is funded by the National Institute of Health Research in the United Kingdom which makes PPI mandatory for all their funded research. In many parts of the western world, citizen activism and full participation is contributing to policy making and governance, not just in healthcare or research but in other governmental decisions.

We also need to consider how to involve patient and public members in clinical care and research decision making. When the British journals started demanding a section on PPI for all research articles that were submitted for publication, there were many researchers here who did not understand what it meant. Neither did I at that time. So comments like ‘it was a qualitative study, we interviewed patients so that was the patient involvement right there’ or ‘we are publishing this paper so that we can share it with the patients and public’ were some of the things that were being said. This is not true involvement. Neither is just ticking yes to the box that says that we did involve patients and the public but not actually doing so.

Sherry Arnstein said that citizen participation is actually about citizen power. It is the redistribution of power that enables the have-not citizens, presently excluded from the political and economic processes, to be deliberately included in the future. Does that make you feel uncomfortable? Perhaps it is because you are in a position of power. Or that you are used to the dynamics and do not like change? In her seminal paper, Sherry describes the 8 rungs on the ladder of citizen participation. At the moment, much of clinical care and research occurs at the bottom rung. I am reminded of the participants in my studies telling me how they were ignored by health care providers during consultations and visits. A general in the army, think of that a general who is obeyed without hesitation by the army, told me how powerless he felt against a medical officer in the emergency unit. We have to find our way from non-participation, up through the degrees of tokenism towards degrees of actual citizen power, where there is partnership, delegated power and actual citizen control. This is the vision of democracy that is government of the people, by the people, for the people. [Lincoln, Gettysburg address] It forms the basis of the Declaration of Helsinki, the right of the individual to self-determination and informed decision making. It requires a a radical modification of the traditional paradigm of healthcare: moving from a vertical “organizational-centric” approach in treating diseases, to a horizontal one designed and tailored on the patient’s expectations and needs. [Palozzi, Schettini and Chirico. Sustainability 2020] Patient centered care is one of the key trends for Malaysia’s transformation plan but are we actually doing this? Or is this just still tokenism or worse, non-participation. Let us consider how many patients or public members are truly involved in our clinical care and research decision making. The truth of it is that the numbers are still dismal. I have already started talks with the FOM and UMMC to move PPI here. And also discussed this with the Institute of Clinical Research Malaysia. We also have patient and public members who have been working with us and the NIHR in UK who are ready to share their skills and experiences.

I started this journey as a patient so it is fitting that i end my journey as a patient. And that is as it should be. Being a patient is no longer a position of inferiority, it should never have been perceived to be that, Instead, it is and should be a position of power and focus where the patient is the individual that guides and direct the decisions pertinent to her well being in all domains be it health or life in general. And i am not defined by my disease as i am not defined by my career or my ethnicity or my religion. I am me, Su May. your friend, colleague, relative, teacher, student, your peer.

I am so thankful that i have had the opportunity to experience so many different roles, student and teacher, patient and doctor, daughter and mother, girlfriend then wife. All these viewpoints, these moments, the journeys have made my life so rich. You have all made my life into a viral Netflix limited series by sharing your lives with me; the students who i saw change from being indifferent and callous to genuine caring competent doctors, the patients who shared their life stories and placed their trust in me, my colleagues who have become part of my family, my friends who have supported me and my family who always always had my back. You have given colour to my world, music to my playlists and flavour to my days. I remember it all, the patients who have passed, the laughter and the tears, the smell of hospital disinfectant and the cold of the wards and Oxford winters. One day it might all be gone. One day i might forget all of it. But for now, let me appreciate all this and tell you that I love you. Thank you for being a part of my life.

